# Assessing the relationship between knowledge and the actual use of contraceptives among childbearing women in South-South Nigeria: evidence from the 2018 Nigeria demographic and health survey

**DOI:** 10.1186/s12889-022-14728-y

**Published:** 2022-11-29

**Authors:** Vitalis U. Ukoji, Princewill O. Anele, Chukwuechefulam K. Imo

**Affiliations:** 1 Department of Sociology, Faculty of Social and Management Sciences, Nigeria Police Academy, Wudil, Kano State Nigeria; 2 Department of Sociology, Adekunle Ajasin University, Akoko-Akungba, Ondo State Nigeria

**Keywords:** Contraceptive, Use, Knowledge, Childbearing women, South-South Nigeria

## Abstract

**Background:**

Nigeria has one of the world's highest fertility rates, which is detrimental to its public health and socioeconomic growth. Despite several efforts by the country and other development partners to reduce high fertility by increasing contraceptive use, the contraceptive prevalence rate among childbearing women remains low, particularly in the South-South compared to other Southern regions. This study, therefore, assessed the relationship between knowledge of and actual use of contraception among women in South-South Nigeria.

**Methods:**

The study employed a cross-sectional analysis of a nationally representative weighted sub-sample of 4,553 South-South childbearing women extracted from the 2018 National Demographic and Health Survey dataset. The dataset was weighted and examined for missing values that were excluded during the analyses at univariate, bivariate, and multivariate levels. The analyses involved a baseline descriptive analysis, a chi-square test, and logistic regression models using Stata software. The results of the explanatory variables were presented as odds ratios (OR) and 95% confidence intervals (CI).

**Results:**

Eighty-two per cent of the respondents knew at least one form of contraception, while approximately 82% never used any contraceptive method. The likelihood of using any contraceptive method increased among those who knew about contraceptives (aOR: 1.40; CI: 0.93–2.11). Also, contraceptive use was significantly higher among women and their partners who had post-primary education (aOR: 1.34; CI: 1.25–2.43 and aOR: 1.74; CI: 1.25–2.43, respectively). Furthermore, the prevalence of contraceptive use among women significantly increased with an increase in the household wealth index. Similar results were recorded among women who had five or more living children, who were residents of Rivers State, were married or lived with their partners, were aged 35 years or older, and were currently working.

**Conclusions:**

Contraceptive knowledge was high but did not translate into actual practice among childbearing women in South-South Nigeria. The use of any contraception was highly influenced by contraceptive knowledge, education, age, marital status, place of residence, and household wealth index, among others. Therefore, some policy issues relating to contraceptive knowledge and actual adoption must be addressed to improve the low rate of contraceptive use in Nigeria.

## Introduction

The global population is expected to reach 8.6 billion by 2030 [1]. Many developing countries are already dealing with the consequences of a large population. Unchecked population growth, in low-resource settings, presents diverse challenges to all and sundry. As a result, many countries put in place a variety of population policies to lower the high fertility rate. A proven strategy for fertility regulation is the effective use of contraceptives [2, 3]. Contraceptive use benefits individuals, families, and society at large [4]. It promotes national and global socioeconomic development, and improves women's reproductive health [3, 5–7]. It reduces the number of unintended pregnancies, and unsafe abortions and lowers maternal mortality [7–10]. Conscious efforts are ongoing to encourage women of reproductive age to use contraception. Research supports a rise in the number of women aged 15–49 using contraceptives globally. For instance, there was a nine-point rise in the global contraceptive prevalence rate (CPR) from 55% in 1990 to 64% in 2015 [7]. A further increase from 663 to 851 million women aged 15–49 who used contraceptives was recorded between 2000 and 2020 [3]. Many developing countries with high fertility rates appear to miss out on this global uptick because of the low use of modern birth control measures [10]. For example, sub-Saharan Africa (SSA) has the lowest modern contraceptive prevalence rate (mCPR) and accounts for 21% of the global total [7, 11].

Similar to SSA, the CPR is low in Nigeria, and efforts to improve it have largely been unsuccessful. In 2012, the Nigerian government, alongside some foreign donors, set an mCPR target of 27% by 2020 [9]. To meet this goal, there were initiatives to increase the availability of family planning services at all levels of healthcare. The target was also aimed at increasing media campaigns to persuade women to seek and accept free access to modern contraception [12–14]. Despite these efforts, Nigeria's CPR among married women in 2018 was 17% for any method and 12% for mCPR [9]. Nigeria's rising population, socioeconomic woes, poor health, and maternal mortality indicators may be attributed in part to the country's inability to improve on its low mCPR. As a result, 48% of sexually active women and 19% of married women aged 15–49 who would otherwise like to avoid pregnancy have an unmet need for modern contraception [9].

In contrast to the low CPR, previous studies in Nigeria [15–18] found high contraceptive knowledge among childbearing women aged 15–49. The low use of contraception in Nigeria can be attributed to individual, family, and community factors. Culture, religion, myths and misconceptions, the number of living children, employment status, lack of a partner's support, and other factors all undermine effective contraceptive use [19–21]. As a result, the low mCPR in Nigeria and other SSA countries may have little to do with a lack of contraceptive knowledge and awareness. Most studies to date have concentrated on the factors that contribute to low contraception use among women aged 15–49 [4, 8, 14, 22]. The relationship between having a high level of knowledge about contraception and using it is not well understood. As a result, this paper aims to fill that gap and push the boundaries of knowledge about contraceptive use in Nigeria and the South-South geopolitical zone even further.

Nigeria recognizes six geopolitical zones, a concept that allows states with comparable socioeconomic, cultural, religious, historical, and political values to be grouped. They are the geopolitical zones of the North-East, North-West, North-Central, South-West, South-East, and South-South. The contraceptive prevalence rate is higher in Nigeria's southern regions on average but lower in the northern regions [9, 23, 24]. For example, it ranges from 2% in the northern states of Yobe and Sokoto to 29% in Lagos State in Nigeria's south [9]. Conservatism, dominant culture, and religion are all documented reasons for the north's low CPR [8, 25]. Low levels of education, a lack of women's autonomy and empowerment, patriarchy, family, and community factors are also issues [4, 26]. Southern Nigerian women, particularly those of Yoruba ethnic origin, are more likely to use contraception [9, 23].

In Nigeria, there is evidence of inter-regional disparities in contraceptive use [7, 27]. However, there have been insufficient attempts to illustrate the intra-regional dynamics of contraceptive knowledge and actual use among specific regions. The various factors at work in the South-South region's low contraceptive uptake are not fully understood. Women in the South-South have more freedom, empowerment, and autonomy than many of their counterparts in the North. The zone also has a high level of knowledge about contraception [23], but the CPR remains inconsistent with these existing realities. Furthermore, despite sharing similar historical, political, social, economic, cultural, and religious contexts, the CPR varies across South-South states. The South-East and South-West zones, which are also located in southern Nigeria, share many socioeconomic similarities with the South-South. The South-South geopolitical zone has the lowest proportion of all women using contraception (20.6%), followed by the South-East (21.0%) and the South-West (26.2%) [9]. However, it differs significantly from the other northern zones, North-Central (13.3%), North-East (8.4%), and North-West (5.7%) [9]. Despite the overlapping circumstances among South-South states, some previous studies on knowledge and use of contraceptives in the region [28–30] and the country at large [31, 32] have failed to examine the relationship between contraceptive knowledge and actual use in South-South Nigeria. As a result, this study looked at the relationship between contraceptive knowledge and actual use among childbearing women in Nigeria's South-South geopolitical region. As a result, policies and programmes may be encouraged to help the South-South region transition from high contraceptive knowledge to actual contraceptive use.

## Methods

### Data source and sample

The 2018 NDHS provided the dataset for this study. It is a cross-sectional survey that is nationally representative of 41,821 women of childbearing age (15–49) and men aged 15–59. The survey collected data on fertility, nursing habits, nutritional status of women and children, awareness of and utilisation of family planning services, and so on. For the 2018 NDHS, a two-stage stratified cluster sampling method was used. Stratification was achieved by categorising the 36 states and the Federal Capital Territory (FCT) into urban and rural areas, yielding 74 sampling strata. Using a probability proportional to size method, 1,400 enumeration areas (EAs) were chosen in the first stage. The second stage was completed by conducting a household listing with the previously selected EAs. By using equal systematic sampling, a predetermined number of 30 households emerged from clusters (EAs) in the second stage. To ensure the representativeness of the data, the 2018 NDHS weighted the sample of childbearing women from each state. Weights were generated to adjust the number of women in each state so that each state's contribution to the total is proportional to the actual population of the state. An expanded methodology for collecting data for the 2018 NDHS was illustrated elsewhere in the 2018 NDHS final reports [9]. The South-South geopolitical zone formed the study area for this study. Hence, this study involved a cross-sectional analysis of the data for the South-South subsample of 4,553 childbearing women aged 15–49. The women included in the sample and analyses were childbearing women whose birth histories were recorded in the five years that preceded the survey (i.e., 2013–2018). Using the Women's Questionnaire, data were collected from eligible women on the topic “knowledge, use, and source of family planning methods” through computer-assisted personal interviewing (CAPI).

## Study variables

### Outcome variable

The outcome variable for this study was the "use of any contraceptive method." Information on the use of any contraceptive method was derived from childbearing women's responses to the question, "Current use of contraceptive by methods." Respondents who reported having used modern, traditional, or folkloric methods were coded "1," while those who did not use any method were coded "0."

### Explanatory variable

The main explanatory variable, "knowledge of contraceptives," was derived from asking women to respond to the question regarding knowledge of any contraceptive method. Hence, women who responded not knowing of any contraceptive were categorised as "no" and coded "1" while those who responded in the affirmative to the knowledge of any contraceptive method were classified as "yes" and coded "2".

Evidence of the significant influence of women's marital status, age, education, employment status, wealth index, religion, and residence, as well as their partners' education, on the use of contraceptives in the literature [33–35], and their availability in the accessed dataset, informed the inclusion of selected covariates. The other covariates included in the analysis were the number of children ever born and the number of living children based on the documentation of their association with contraceptive use [36, 37]. Some of the covariates were reclassified from their original categories in the dataset during the analyses for easier presentation and better understanding. For instance, age was regrouped as 15–24, 25–34, and 35 years and above. Education was classified as "no education," "primary education," "secondary education," or "tertiary education." The household wealth index was measured as poor, middle, and rich. Religion was regrouped as Catholic, other Christian, and Islam/others. The number of children ever born and the number of living children, which ranged from "0" to "14" and "0" to "11," respectively, were categorised as "0," "1–4 children," and "5 children and above.

## Data analysis

The dataset was weighted with the appropriate sampling weights as per the Demographic and Health Survey (DHS) sampling scheme to adjust for under- and over-reporting. It was also carefully checked for missing values that were excluded using Stata software (version 15.0). The *svyset* command was used to describe the data sets as products of the survey. The analyses were conducted at three levels, including univariate, bivariate, and multivariate. The baseline descriptive analysis using summarised percentages to describe the study population was used at the univariate level. The Pearson chi-square test was adopted at the bivariate level to examine the association between the outcome variable (use of any contraceptive) and the explanatory variables. At the multivariate level, two different models were fitted to measure the odds ratio (OR) of the association between the use of any contraceptive method and the main explanatory variable (knowledge of contraceptives), as well as the covariates. Model 1 presented the unadjusted logistic regression results, while Model 2 adjusted for all the explanatory variables. In Model 2, all the explanatory variables were fitted because of their significant relationships with the outcome variable. However, marital status was omitted during the analysis because of collinearity. The results of the explanatory variables were expressed as OR with 95% confidence intervals (CI), and the possible association between each of the explanatory variables and the outcome variable was assessed by observing the *p*-values.

## Results

### Sociodemographic characteristics of study population

Table 1 shows that 54.27% of respondents were married and lived with their partners. This was followed by the unmarried (never in a union) group (37.40%). The proportion of respondents who were widowed, divorced, or separated was the lowest in the study population; those aged 25–34 made up 31.74%, and those aged 35 or older made up 33.56%. Nearly 63.5% had a secondary education, and 14.60% had a post-secondary education. Sixteen per cent had received primary education, while 5.03% had no formal education. A little more than 46% had 1–4 children, 33.52% had no children, and nearly 20% had five or more.

**Table 1:** Sociodemographic characteristics of respondents (*N* = 4,553)

Variables	Frequency	Percentage
Current Marital Status		
Never in union	1,703	37.40
Married/living with a partner	2,471	54.27
Widowed/Divorced/Separated	379	8.33
Age		
15–24	1,580	34.70
25–34	1,445	31.74
35 and above	1,528	33.56
Highest educational level		
No education	229	5.03
Primary	759	16.67
Secondary	2,892	63.52
Higher	673	14.78
Husband/partner’s education level		
No education	80	3.23
Primary	397	16.07
Secondary	1,485	60.10
higher	509	20.60
Currently working		
No	1,272	27.94
Yes	3,281	72.06
Wealth index		
Poor	675	14.83
Middle	1,131	24.84
Rich	2,747	60.33
Religion		
Catholic	456	10.02
Other Christians	3,808	83.63
Islam and others	289	6.35
Place of residence		
Urban	1,647	36.17
Rural	2,906	63.83
State of residence		
Edo	605	13.29
Cross River	644	14.14
Akwa Ibom	829	18.21
Rivers	1,060	23.28
Bayelsa	706	15.51
Delta	709	15.57

Almost two-thirds (72.06%) of respondents were currently employed, 27.94% were unemployed, and 60.33% were in the highest wealth quintile. Only 14.83% were in the lowest wealth quintile, while 24.84% were in the middle wealth quintile. Christians—both Catholics (10.02%) and other Christian denominations (83.63%) outnumbered Muslims and other forms of religion (6.35%). Nearly 63.83% of respondents lived in rural settings rather than urban areas (36.17%). Finally, Rivers State had the highest proportion of respondents (23.28%), followed by Akwa Ibom State (18.21%). Delta State (15.57%) and Bayelsa State (15.51%) followed with nearly identical proportions of respondents. The states of Edo (13.29%) and Cross River (14.14%) had the fewest respondents.

## Knowledge and use of contraceptives by methods

Table 2 depicts the overall contraceptive knowledge versus the lack of contraceptive knowledge. In total, just over 82% of study participants were aware of at least one contraceptive method, while nearly 18% were unaware of any contraceptive methods. Despite having a high level of knowledge about contraception, childbearing women in Nigeria's South-South geopolitical zone do not use it. Data show that 81.70% of respondents do not use any form of contraception while just about 18% admitted using any kind of contraceptive method. About 14.06% of those surveyed admitted to using modern contraception methods. Traditional contraceptives, on the other hand, were used by 4.24% of those surveyed.

**Table 2:** Knowledge and use of any contraceptive method in the South-South Geopolitical Zone (*N* = 4,553)

Variables	Frequency	Percentage
Knowledge of contraception		
No	818	17.97
Yes	3,735	82.03
Use of any contraceptive method		
No	3,720	81.70
Yes	833	18.30
Current use by method		
No method	3,720	81.70
Traditional/folkloric methods	193	4.24
Modern methods	640	14.06

## Contraceptive use by States in the South-South Geopolitical Zone, NDHS 2018

**Fig. 1:**
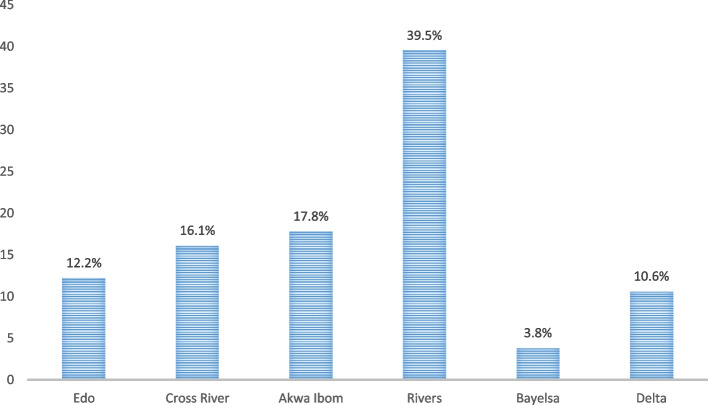
Contraceptive use by States in South-South Geopolitical Zone

Contraceptive use among childbearing women across the six states in the South-South is illustrated below. As shown in Fig. 1, Rivers and Bayelsa States had the highest and lowest rates of contraceptive use, respectively. Rivers State, in particular, had the highest rate of contraceptive use (39.5%). In terms of contraceptive use, Akwa Ibom State (17.8%) and Cross River State (16.1%) ranked second and third, respectively. Bayelsa State (3.8%) and Delta State (10.6%) had the lowest prevalence of contraceptive use among South-South states.

## The most common contraceptives among women of reproductive ages in the South-South region

**Fig. 2:**
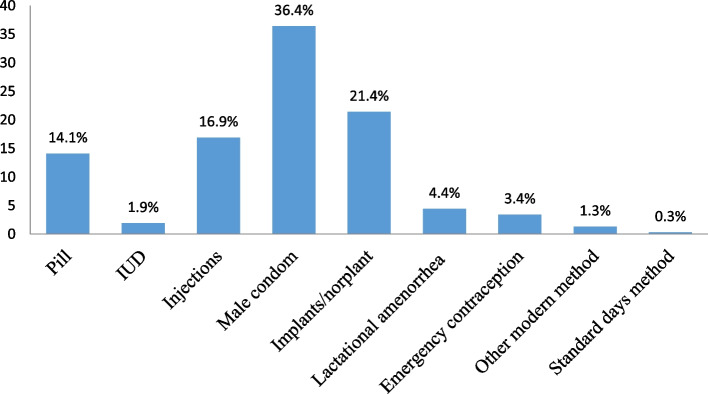
Most common types of contraceptives in South-South Geopolitical Zone

Figure 2 depicts the most common types of contraception among reproductive-age women in the South-South geopolitical zone. Pills, intrauterine devices (IUDs), injections, male condoms, implants/Norplant, lactational amenorrhea, emergency contraception, and the standard-days-methods are among the various forms of contraception. Male condoms (36.4%), implants/Norplant (21.4%), injections (16.9%), and pills (14.1%) remained the most commonly used modern contraceptives among sexually active childbearing women in the South-South zone. In contrast, the standard-days method (0.3%), IUD (1.9%), emergency contraception (3.4%), and lactational amenorrhea (4.4%) were the contraceptive methods that were least mentioned.

## Bivariate association of contraceptive use with all the explanatory variables

Table 3 shows the results of the bivariate analysis of the test of associations between the outcome variable (use of any contraceptive methods) and the explanatory variables. Data show a significant association between all explanatory variables, except marital status (X^2^ = 12.8297; *p* = 0.002), type of place of residence (X^2^ = 5.6025; *p* = 0.018), and use of any contraceptive method. The Chi-square statistics show that the use of any contraceptive method was significantly associated with women’s knowledge of contraceptive methods (X^2^ = 95.0778; *p* = 0.000). Here, knowing about contraceptives was related to the use of any contraceptive methods. A similar result was found for age, as a woman's age and use of a contraceptive method were found to be significantly related (X^2^ = 62.5074; *p* = 0.000). Likewise, education was significantly related to the use of any contraceptive method (X^2^ = 51.9577; *p* = 0.000). In this case, higher education was more related to the use of any contraceptive methods than lower education, which is significantly related to the non-use of contraceptives. The link between women's education and contraceptive use extended to their husbands’/partners' education (X^2^ = 32.5770; *p* = 0.000). In this case, women with more educated husbands/partners were more likely to use any contraceptive method than their counterparts with less educated husbands/partners. Work status (X^2^ = 34.4654; *p* = 0.000), wealth index (X^2^ = 29.2398; *p* = 0.000), religion (X^2^ = 34.3295; *p* = 0.000), and use of any contraceptive methods all had significant associations. Similar findings were found for the number of children ever born (X^2^ = 27.7415; *p* = 0.000), the number of children alive (X^2^ = 30.8104; *p* = 0.000), and the use of any contraceptive methods. While working women were more likely to use any form of contraception, those who did not work were more likely to ignore the use of contraception. Similarly, women with a higher wealth index were more likely to use any form of contraception than women with a lower wealth index. Regarding religion, we found that Christians were more likely to use any form of contraception than Muslims. Women who birthed five or more children were also more likely to adopt any contraceptive method than those with fewer children. The same applied to women with a higher number of children alive than those with a lower number of children alive.

**Table 3:** Unadjusted bivariate analysis of contraceptive use in South-South Nigeria

Background characteristics	Current use of contraceptives		X^2^ (*p*-Value)
	No method	Used any method	
Knows contraceptive methods			
No	766 (20.59%)	52 (6.24%)	95.0778 (0.000)
Yes	2,954 (79.41%)	781 (93.76%)	
Marital status			
Never in union	1,433 (38.52%)	270 (32.41%)	12.8297 (0.002)
Married	1,973 (53.04%)	498 (59.78%)	
Widowed/divorced	314 (8.44%)	65 (7.81%)	
Age			
15–24	1,379 (37.07%)	201 (24.13%)	62.5074 (0.000)
25–34	1,101 (29.60%)	344 (41.30%)	
35 and above	1,240 (33.33%)	288 (34.57%)	
Highest education			
No education	215 (5.78%)	14 (1.68%)	51.9577 (0.000)
Primary	640 (17.20%)	119 (14.29%)	
Secondary	2,366 (63.60%)	526 (63.14%)	
Higher	499 (13.41%)	174 (20.89%)	
Husband/partner’s educational level			
No education	79 (4.00%)	1 (0.20%)	32.5770 (0.000)
Primary	342 (17.33%)	55 (11.05%)	
Secondary	1,156 (58.59%)	329 (66.06%)	
Higher	396 (20.07%)	113 (22.69%)	
Currently working			
No	1,108 (29.78%)	164 (19.69%)	34.4654 (0.000)
Yes	2,612 (70.22%)	669 (80.31%)	
Wealth index combined			
Poor	588 (15.81%)	87 (10.44%)	29.2398 (0.000)
Middle	954 (25.65%)	177 (21.25%)	
Rich	2,178 (58.55%)	569 (68.31%)	
Religion			
Catholic	362 (9.73%)	94 (11.28%)	34.3295 (0.000)
Other Christians	3,085 (82.93%)	723 (86.79%)	
Islam and others	273 (7.34%)	723 (1.93%)	
Place of residence			
Urban	1,316 (35.38%)	331 (39.74%)	5.6025 (0.018)
Rural	2,404 (64.62%)	502 (60.26%)	
Children ever born			
0	1,310 (35.22%)	216 (25.93%)	27.7415 (0.000)
1–4	1,705 (45.83%)	422 (50.66%)	
5 and above	705 (18.95%)	195 (23.41%)	
Number of living children			
0	1,336 (35.91%)	218 (26.17%)	30.8104 (0.000)
1–4	1,786 (48.01%)	444 (53.30%)	
5 and above	598 (16.08%)	171 (20.53%)	

## Multivariate analysis of the association between explanatory and contraceptive use

Table 4 shows the results of the multivariate analysis. The unadjusted logistic regression results in Model 1 showed that women who knew about contraceptives were significantly more likely to use contraceptives (OR: 3.90; CI: 2.91–5.21) compared to those who reported no knowledge of the contraceptive methods. Also, the likelihood of using any contraceptive method significantly increased among married women or those living with their partners (OR: 1.34; CI: 1.14–1.58. Older women aged 25–34 years and 35 years and older were respectively 2.14 times and 1.59 times more likely to use any contraceptive method than their counterparts aged 15–24. Concerning education, the results showed that the likelihood of using any contraceptive method was significantly higher among women (OR: 1.57; CI: 1.29–1.92) and their partners (OR: 2.14; CI: 1.59–2.88) with post-primary education compared with those in the reference categories. The likelihood of women using any form of contraception increased significantly as the number of children born increased. Women with 1–4 children and those with five or more children, for instance, were 1.50 and 1.68 times more likely to use any contraceptive method than those without children.

**Table 4:** Unadjusted and adjusted logistic regression analysis of contraceptive use by knowledge of contraceptive and covariates, NDHS 2018

	Model 1	Model 2
Variable/Category	OR (95% CI)	aOR (95% CI)
Knowledge of contraceptives		
No (Ref.)	1.00	1.00
Yes	3.90 (2.91–5.21)***	1.40 (0.93–2.11)
Marital status		
Never in union (Ref.)	1.00	-
Married/living with a partner	1.34 (1.14–1.58)***	-
Widowed/divorced/separated	1.10 (0.82–1.48)	-
Age		
15—24 (Ref.)	1.00	1.00
25 – 34	2.14 (1.77–2.60)***	1.05 (0.72–1.53)
35 and above	1.59 (1.31–1.94)***	0.87 (0.58–1.30)
Educational attainment		
No education/primary (Ref.)	1.00	1.00
Secondary/tertiary	1.57 (1.29–1.92)***	1.34 (1.00–1.78)*
Partner's educational attainment		
No education/primary (Ref.)	1.00	1.00
Secondary/tertiary	2.14 (1.59–2.88)***	1.74 (1.25–2.43)**
Children ever born		
0 (Ref.)	1.00	1.00
1—4 Children	1.50 (1.25–1.80)***	0.47 (0.11–1.99)
5 children and above	1.68 (1.35–2.07)***	0.42 (0.09–1.91)
Number of living children		
0 (Ref.)	1.00	1.00
1—4 Children	1.52 (1.28–1.82)***	3.24 (0.77–13.70)
5 children and above	1.75 (1.40–2.19)***	4.10 (0.90–18.65)
Currently working		
No (Ref.)	1.00	1.00
Yes	1.73 (1.44–2.08)***	1.16 (0.85–1.59)
Wealth index		
Poor (Ref.)	1.00	1.00
Middle	1.25 (0.95–1.65)	1.55 (1.04–2.32)*
Rich	1.77 (1.38–2.25)***	1.87 (1.28–2.73)**
Religion		
Catholic (Ref.)	1.00	1.00
Other Christians	0.90 (0.71–1.15)	0.92 (0.66–1.27)
Islam & others	0.23 (0.13–0.39)***	0.54 (0.27–1.06)
Place of residence		
Urban (Ref.)	1.00	1.00
Rural	0.83 (0.71–0.97)*	0.94 (0.74–1.19)
State of origin		
Edo (Ref.)	1.00	1.00
Cross River	1.05 (0.78–1.41)	1.05 (0.72–1.54)
Akwa Ibom	1.05 (0.79–1.39)	1.04 (0.71–1.52)
Rivers	2.59 (2.02–3.32)***	1.63 (1.18–2.24)**
Bayelsa	0.18 (0.12–0.29)***	0.17 (0.10–0.29)***
Delta	0.72 0.53–0.97)	0.68 (0.47–1.00)

*p < 0.05; **p < 0.01; ***p < 0.001; Ref. = reference category

Similarly, the likelihood of using any contraceptive method significantly increased among women who had 1–4 and 5 or more living children (OR: 1.52; CI: 1.28–1.82 and OR: 1.75; CI: 1.40–2.19, respectively). Women who reportedly have a current job had higher odds of using any contraceptive method (OR: 1.73; CI: 1.44–2.08) than those who were unemployed. An increase in household wealth status positively influenced the use of any contraceptive method among women. Women from middle- and rich household wealth statuses were 1.25 times and 1.77 times more likely to use any contraceptive method compared with their counterparts from poor households. Concerning religion, the results showed that the likelihood of using any contraceptive method significantly reduced among women of Islam and traditional religious backgrounds (OR: 0.23; CI: 0.13–0.39). Also, the likelihood of using any contraceptive method was significantly reduced among women who were found in rural areas (OR: 0.83; CI: 0.71–0.97) compared to urban residents. The results further showed that the likelihood of using any contraceptive methods significantly increased among women who reported to have come from Rivers State (OR: 2.59; CI: 2.02–3.32) but reduced among their counterparts from Bayelsa State (OR: 0.18; CI: 0.12–0.29) compared to those from Edo State.

The adjusted logistic regression results are presented in Table 4, Model 2. Although not significant, the results showed that the likelihood of using any contraceptive method was higher among women who knew about contraceptives (aOR: 1.40; CI: 0.93–2.11), compared with those in the reference category. The likelihood of using any contraceptive method was significantly higher among women and their partners with post-primary education (aOR: 1.38; CI: 1.00–1.78; and aOR: 1.74; CI: 1.25–2.43, respectively) than those in the reference category. The results further showed that an increase in the household wealth index positively influenced the likelihood of using any form of contraception among women. For instance, women from middle- and high-wealth indexes were (1.55 times and 1.87 times, respectively, more likely to use any contraceptive method than those in poor households. The use of any contraceptive method significantly increased among women from Rivers State (aOR: 1.63; CI: 1.18–2.24) and decreased among women from Bayelsa (aOR: 0.17; CI: 0.10–0.29) compared to those in the reference category.

## Discussion of findings

Using data from the 2018 NDHS, this study examined contraceptive knowledge and the actual use among childbearing women aged 15–49 in Nigeria's South-South geopolitical zone. The women in the sample were those whose birth histories had been documented in the five years preceding the survey (i.e. 2013–2018). They responded to questions about the 2018 NDHS family planning services awareness and use module. In all, findings showed that most childbearing women in Nigeria's South-South region had high knowledge about contraception. There are several possible explanations for this high level of contraceptive knowledge in the South-South. Government and other non-governmental organisations fund many local radio and television programmes promoting family planning services. They are also advertised in newspapers, magazines, and at prenatal and postnatal clinics in hospitals [24]. Furthermore, women in Nigeria's south are well-informed and exposed [37], and thus open to using contraception. Previous research has found that childbearing women in southern Nigeria [17, 18, 27], North-West Nigeria [16], and Uganda [38] have comparable or even higher knowledge of contraceptives.

The level of contraceptive knowledge influences increased contraception demand and adoption [18, 31]. Our findings showed that, despite widespread contraceptive knowledge among childbearing women, actual contraceptive use was lacking in our study population. Given that women in the South-South have a medium to a high level of education [18], this finding is somewhat surprising. Women with higher levels of education are more likely than their counterparts with lower levels of education to use contraception [24, 39, 40]. The fact that few respondents have ever used any form of contraception emphasizes the gap between respondents' knowledge and actual contraception use. It underscores that, in addition to individual-level factors, family- and community-level factors are critical to contraceptive use [24, 41]. For example, a woman who is aware of the benefits of contraception may not use it due to the influence of an unwilling partner, religious dogma, or other social circumstances. Regardless of the disparity between widespread contraceptive knowledge and low contraceptive use, the bivariate results showed that women with more contraceptive knowledge were more likely to use any contraception methods. Furthermore, the unadjusted multivariate analysis in Model 1, Table 4 suggested that childbearing women with contraceptive knowledge had a higher probability of using any contraceptive method than those with no contraceptive knowledge. In line with our findings, there are additional reports of discrepancies in Nigeria between high contraceptive knowledge and low contraceptive use [17, 27, 37, 42]. Aside from the low contraceptive use, a study [43] found a decrease in contraceptive use, which has implications for maternal and child health. The low CPR implies Nigeria has missed the 27% CPR target set for 2020. Hence, it appears Nigeria is trapped in the knowledge stage rather than the practice stage. A cocktail of social, economic, cultural, and religious issues may contribute to the failure. Additional research is needed to determine how these elements interact to stall the transition from high-level contraceptive knowledge to high CPR in South-South Nigeria.

Rivers State was far ahead of the other South-South states, ranking nearly twice as high as Akwa Ibom. Rivers State reported a significantly higher figure than Bayelsa and Delta States. Contraceptive prevalence rate disparities in the South-South could be explained by differences in education, urbanisation, and income levels within these states. Rivers State, for example, is the most populous and urbanised state in the South-South geopolitical zone. Its citizens are also more likely to be educated and have more control over reproductive decisions. This may play a role in Rivers State’s comparatively high contraceptive prevalence rate. Furthermore, multivariate results revealed that women in Rivers State were more likely than women from other states in the zone to use any form of contraception. Previous research has shown that women in southern Nigeria, including Rivers State, use more contraception [23, 36]. The prevalence of contraceptive use in the South-South reflects that of the national level. Previous studies [9, 44] confirmed Nigeria's low national and subnational contraceptive prevalence rates.

As previously stated, the male condom was the most popular method of contraception among women in the South-South. Women were also big on implants, injections, and pills, in addition to male condoms. The IUD and the standard-days method, on the other hand, were among the least popular approaches. Given its low cost and ease of use, it is unsurprising that the male condom has become so popular. This differs from other techniques, which may necessitate invasive surgical procedures or a certain level of proficiency. The finding on the male condom being the most widely available type of contraception is supported by previous research that found similar results [16, 45, 46].

Further, bivariate and multivariate analyses revealed significant influences on contraceptive use. The bivariate results suggest that knowing about contraception, age, education (both women and their husbands/partners), employment, wealth index, and religion were all associated with the use of any contraceptive methods. Marital status and rural/urban residence were unimportant factors influencing contraceptive use. However, the adjusted logistic regression results (Model 2, Table 4) indicated a significant influence on marital status and rural/urban residence. In essence, multivariate results revealed that women who had contraceptive knowledge, were married/and living with partners, were older, had post-primary education, or whose partners had post-primary education were more likely to use any contraceptive methods. Other factors that influenced contraceptive use include having more living children, being currently employed, having a higher wealth index, being non-Muslim, and living in cities. Age has a significant impact on contraceptive use. In essence, older women are more likely to use modern contraception than younger women. This pattern is possible because older women may have reached their desired family size and may turn to contraception to avoid further pregnancies [21, 47, 48]. Overall, increased education empowers women to work, earn an income, take control of their lives, and make informed decisions about whether and how to use contraception. Previous research from Nigeria [37, 46] and Senegal [49] supports our findings. However, one Indonesian study [50] contradicted our finding on the role of a higher level of education in the use of contraception.

According to previous research, women from the wealthiest households were more likely to use contraception than those from the poorest households [38, 51, 52]. The affordability of contraception and the decision-making autonomy of wealthier women easily explain why they use contraception more than their poorer counterparts [53]. Working women, on the other hand, are more likely to use contraception than the unemployed. A plausible explanation is that women who prioritise career growth and professional development may prefer to avoid any pregnancy-related interferences with their career development. Previous research has also confirmed the impact of religion on contraceptive use. In line with previous findings [23, 35, 53], Christians were more likely to use contraception than Muslims. While modern Christianity frowns at multiple partners, Islam and traditional religions accommodate such. In this case, Christians are likely to use contraception to limit their number of children, especially since there is no competition for children among co-wives. By contrast, there may be a desire for many children among Muslim co-wives, as Islam views multiple births as a blessing from God.

## Strengths and limitations

The dataset's representativeness is a strength of this study. The NDHS is a national cross-sectional survey that collects information from a sample of people all over the country. As a result, it allows for some safe generalisations. Furthermore, strict procedures are implemented while collecting data to avoid sampling bias and data errors. Regardless of its strengths, this study may have limitations. Particularly, the nature of the NDHS means it is a snapshot of attitudes or events among respondents. Also, using secondary data implies that some important nuances are lacking since qualitative insights are not well captured except for a few open-ended responses. Furthermore, there is a possibility of misreporting contraceptive use due to forgetfulness. Respondents also have a proclivity for socially desirable responses. Despite these potential limitations, the findings are significant for evidence-based policies and interventions aimed at transitioning from contraceptive knowledge to actual use.

## Conclusions

The study examined contraceptive knowledge and use among childbearing women in South-South Nigeria. The most common types of contraceptives, women's knowledge of various contraceptives, and the relationship between the key explanatory factors and contraceptive use were all considered. The government and other non-governmental organisations run programmes that promote family planning services through the use of contraception. Doubtless, the result is that childbearing women throughout Nigeria, including the South-South geopolitical zone, are well aware of contraception. This study shows that, despite widespread knowledge generated by the ongoing effort to use contraception, actual practice has yet to follow. Except for Rivers State, states in the South-South had low contraceptive use during the survey years. Furthermore, the study found that contraceptive use is associated with education level, age, wealth quintile, being married and living with a partner, number of children alive, and number of children ever born, among other factors. As a result, if the South-South's low CPR is to improve, the issues that impede translating high contraceptive knowledge into actual use must be addressed head-on. This could reduce Nigeria's high fertility rate and improve the country's public health and socioeconomic challenges. It may also aid in meeting the SDG target of improving maternal and child health. Hence, more strategies and initiatives should be developed to encourage childbearing women in Nigeria's South-South to use contraception. These strategies should be developed in light of the factors at the family, community, and societal levels that discourage childbearing women from moving from high contraceptive knowledge to actual use.

## Data Availability

The DHS Programme archive is a third-party database and is open to the public and can be accessed at https://dhsprogram.com/data/dataset_admin/index.cfm. To access the survey data used in this study, there is a need to register and a request made to the ICF, DHS programme.

## Abbreviations

CPR: Contraceptive Prevalence Rate
mCPR: Modern Contraceptive Prevalence Rate
SSA: Sub-Saharan Africa
STIs: Sexually Transmitted Infections
DHS: Demographic and Health Surveys
NDHS: Nigeria Demographic and Health Survey
NPC: National [Nigeria] Population Commission
FCT: Federal Capital Territory
USAID: United States Agency for International Development
WHO: World Health Organisation
EAs: Enumeration Areas
IUD: Intrauterine Device
NHREC: National Health Research Ethics Committee of Nigeria
SDGs: Sustainable Development Goals
